# Restoration-repair potential of resin-modified glass ionomer cement

**DOI:** 10.1590/1807-3107bor-2024.vol38.0076

**Published:** 2024-07-23

**Authors:** Carolina Lopes da Silva, Cleber Paradzinski Cavalheiro, Cassiane Gonçalves de Oliveira da Silva, Daniela Prócida Raggio, Luciano Casagrande, Tathiane Larissa Lenzi

**Affiliations:** (a) Universidade Federal do Rio Grande do Sul - UFRGS, School of Dentistry, Post-Graduate Program in Dentistry, Porto Alegre, RS, Brazil.; (b) Universidade Federal do Rio Grande do Sul - UFRGS, School of Dentistry, Porto Alegre, RS, Brazil.; (c) Universidade de São Paulo – USP, School of Dentistry, Department of Orthodontics and Pediatric Dentistry, São Paulo, São Paulo, Brazil

**Keywords:** Composite Resins, Dental Restoration Repair, Glass Ionomer Cements

## Abstract

This in vitro study aimed to evaluate the repair bond strength of resin-modified glass ionomer cement using either the same material or a universal adhesive in the etch-and-rinse and self-etch modes plus resin composite. Twenty-four resin-modified glass ionomer cement blocks were stored in distilled water for 14 d and thermocycled. Sandpaper ground specimens were randomly assigned to three experimental groups according to the repair protocol: resin-modified glass ionomer cement (Riva Light Cure, SDI) and universal adhesive (Scotchbond Universal Adhesive, 3M Oral Care) in etch-and-rinse or self-etch modes and nanohybrid resin composite (Z350 XT, 3M Oral Care). After 24 h of water storage, the blocks were sectioned, and bonded sticks were subjected to the microtensile bond strength (μTBS) test. One-way ANOVA and Tukey's test were used to analyze the data. The failure mode was descriptively analyzed. The highest μTBS values were obtained when the resin-modified glass ionomer cement was repaired using the same material (p < 0.01). In addition, the mode of application of the universal adhesive system did not influence the repair bond strength of the resin-modified glass ionomer cement. Adhesive/mixed failures prevailed in all groups. Repair of resin-modified glass ionomers with the same material appears to be the preferred option to improve bond strength.

## Introduction

Resin-modified glass ionomer cements are commonly used to restore primary teeth and non-carious cervical lesions in the permanent teeth. The annual failure rate of these restorations is approximately 2.7%^1^ in non-carious cervical lesions, and varies from 0.6–16.9% in primary teeth.^2^ When restorative reintervention is needed, repair is considered a more conservative approach to replacement.^3^ Restoration repair has gained increasing acceptance among dental practitioners, especially in cases of marginal defects, partial loss or fracture of the restoration, and margin repair due to carious lesions.^4^ Most dentists reported performing resin composite restoration repairs, whereas the proportion of repaired glass-ionomer cement restorations is low.^4^ Although clinicians prefer repairing a restoration with the same material,^4^ repair protocols are inconsistent, making decisions to replace or repair defective restorations difficult.

Few studies^5–7^ have assessed the repair potential of resin-modified glass ionomer cements with resin-modified glass ionomer cement or resin composite, and the results are contradictory. Furthermore, as repair may occur sometime after the restoration’ placement, the aging of restorative material is important. Only one study^7^ simulated the aging of the glass ionomer cement surface prior to repair protocol.

Therefore, this study sought to evaluate the repair bond strength of aged resin-modified glass ionomer cement using either the same material or a universal adhesive in etch-and-rinse and self-etch modes *plus* resin composite.

## Methods

This study followed the CRIS Guidelines for *in vitro* studies, as discussed in the 2014 concept note.^8^

The following materials were tested: encapsulated resin-modified glass ionomer cement (Riva Light Cure, A1 and A3 shades; SDI, Bayswater, Australia), nanohybrid resin composite (Filtek Z350 XT, A1B shade; 3M Oral Care, Saint Paul, USA), and a universal adhesive system (Scotchbond Universal Adhesive, 3M Oral Care, Saint Paul, USA) in etch-and-rinse and self-etch modes. A detailed description of the materials used is provided in Table 1.

**Table 1:** Main composition and manufacturer's instructions of the materials used.

Material	Main components	Repair protocol
Riva Light Cure	Etchant: 37% phosphoric acid	Apply the etchant for 20 s
A1 and A3 shades (SDI, Bayswater, Victoria, Australia)		Rinse thoroughly with water
	Compartment 1: Acrylic acid homopolymer (15–25%), 2-hydroxyethyl methacrylate (15–25%), dimethacrylate cross-linker (10–25%), acid monomer (10–20%), tartaric acid (5–10%)	Remove excess water
	Compartment 2: Glass powder (93–100%)	Activate the capsule by pushing the plunger until it is flush with the body
		Place the capsule into the amalgamator for 10 s
		Place the capsule into the Riva applicator
		Click the trigger of the applicator until glass ionomer paste is seen through the clear nozzle
		Insert the material in 2 mm increments
		Light cure for 20 s each increment
		Apply petroleum jelly
Scotchbond Universal Adhesive (3M Oral Care, St. Paul, USA)	Etchant: 37% phosphoric acid	Self-etch mode
		Apply the adhesive for 20 s with vigorous agitation
	MDP phosphate monomer, dimethacrylate resins, HEMA, methacrylate-modified polyalkenoic acid copolymer, filler, ethanol, water, initiators, silane	Gentle air thin for 5 s
		Light cure for 10 s
		Etch-and-rinse mode
		Apply the etchant for 15 s
		Wash and totally dry the surface
		Apply the adhesive as the self-etch mode
Resin composite Z350 XT	Bis-GMA, UDMA, TEGDMA, Bis-EMA, non-agglomerated/non-aggregated 20 nm silica filler, non-agglomerated/non-aggregated 4 to 11 nm zirconia filler, and aggregated zirconia/silica cluster filler	Insert the resin composite in 2 mm increments
A1 shade (3M Oral Care, St. Paul, USA)		Light cure for 20 s each increment
	

MDP: 10-methacryloyloxydecyl-dihydrogen-phosphate; Bis-GMA: bisphenyl-glycidyl methacrylate; HEMA: 2-hydroxyethyl methacrylate; TEGDMA: triethylene glycol dimethacrylate; Bis-EMA: ethoxylated bisphenol-A dimethacrylate; UDMA: urethane dimethacrylate

### Sample size calculation

The sample size was calculated using www.sealedenvelope.com. According to a previous study,^5^ repair bond strength means of resin-modified glass ionomer cement using the same material or resin composite were 2.9 and 15.9 MPa, respectively. Considering a standard deviation of outcome of 9.0 MPa between the experimental groups, using a significance level of 5%, a power of 80% and a two-sided test, the minimum sample size was 8 teeth per group.

### Preparation of aged resin-modified glass ionomer cement blocks

Twenty-four blocks of encapsulated resin-modified glass ionomer cement (Riva Light Cure, A3 shade; SDI, Bayswater, Australia), measuring 8 × 8 mm in depth and width and 4 mm in height, were fabricated using a metallic mold (8 × 8 × 8 mm). The mold was fixed to a glass slab. Each block was made using only one encapsulated resin-modified glass ionomer cement. First, the capsule was activated by pushing the plunger until it was flush with the body. It was then immediately placed in an amalgamator (Ultramat 2, SDI, Bayswater, Australia) and mixed for 10 s. The capsule was removed and placed in a Riva applicator (SDI; Bayswater, Australia). The trigger of the applicator was pressed, and encapsulated resin-modified glass ionomer cement was inserted into the metallic mold in two increments of 2 mm, each of which was light-cured for 20 s using a light-emitting diode curing unit (Radii-cal; SDI, Australia) with a light output of at least 1.250 mW/cm^2^. Light intensity was measured using a radiometer (Demetron Curing, Kerr, Orange, USA). After setting, the resin-modified glass ionomer cement blocks were gently removed from the mold, and the thickness of each block was confirmed using a digital caliper (Absolute Digimatic, Mitutoyo, Tokyo, Japan). The specimens were coated with petroleum jelly and stored in distilled water at 37°C for 14 d prior to aging.^9^ The blocks were further aged by thermal cycling 5000 times between 5°C and 55°C, with a dwell time of 20 s and a transfer time of 3 s.^9^ The aged specimen surfaces were wet-ground with 320-grit silicon carbide grinding paper for 60 s to create standardized repair surfaces, corresponding to those obtained by medium diamond bur grinding.^9^

### Bonding procedures

The 24 aged blocks were randomly assigned (Random Allocation software, version 1.0, Iran) into three experimental groups according to the repair protocol (n = 8): use of encapsulated resin-modified glass ionomer cement, use of universal adhesive in the self-etch mode + nanohybrid resin composite, or universal adhesive in the etch-and-rinse mode + nanohybrid resin composite. Randomization was performed by a staff member who was not involved in any of the laboratory phases. Allocation concealment was guaranteed by using sequentially numbered individual containers that prevented the operator from seeing the blocks before treatment. The aged blocks were carefully placed over the original mold and repaired using encapsulated resin-modified glass ionomer cement (A1 shade) or nanohybrid resin composite (A1B shade). Both materials were applied in two incremental layers, each light-cured for 20 s. This process resulted in 8-mm high specimens. After removal from the mold, the specimen surfaces covered by the mold were cured for 20 s. The repaired blocks were stored individually in distilled water at 37°C for 24 h before testing. A single trained operator performed all procedures.

### Microtensile bond strength (μTBS)

Each composite block was numbered according to the randomization sequence to ensure the blinding of the testing machine operator. Blocks were sectioned into sticks with a cross-sectional area of approximately 0.8 mm^2^ using a water-cooled diamond saw in a cutting machine (Isomet, Buehler, Lake Bluff, USA). The sticks were carefully examined under a stereomicroscope at 40× magnification, and those with interfacial flaws, gaps, bubbles, or other defects were excluded. The cross-sectional area of each stick was measured using a digital caliper (Absolute Digimatic, Mitutoyo, Tokyo, Japan) to calculate the bond strength values, measured in MPa.

The bonded sticks were individually attached to a universal testing machine for microtensile testing (EZ-SX series, Shimadzu Corp., Kyoto, Japan) with cyanoacrylate, and tested at a crosshead speed of 1 mm/min. The μTBS, measured in MPa, was obtained by dividing the load at failure (N) by the cross-sectional area (mm^2^) of each stick.

### Failure mode

A blinded examiner evaluated the mode of failure. The fracture surfaces were examined under a stereomicroscope at 40× magnification to determine the failure mode: mixed/adhesive (failure at the adhesive interface) or cohesive (failure exclusively within the aged resin-modified glass ionomer cement or repair material). Representative specimens from each group were gold-sputtered and analyzed using scanning electron microscopy (SEM) in the secondary electron mode at 10 kV. Premature failure was considered a pre-testing failure owing to specimen preparation.

### Statistical analysis

The block was used as the experimental unit. The μTBS values from every stick from the same block were averaged for statistical analysis. The mean μTBS for every testing group was expressed as the average of the eight blocks used per group. Specimens with cohesive failures were excluded from the data analysis. Premature failures were included in the statistical analysis considering a value of 0 MPa.^10^ Normal data distribution was confirmed using the Kolmogorov-Smirnov test. μTBS means were analyzed using one-way ANOVA and Tukey's *post-hoc* tests. The significance level was set at 5%. Statistical analyses were performed using Minitab18 software (Minitab Inc., State College, USA).

## Results

**Figure 1:**
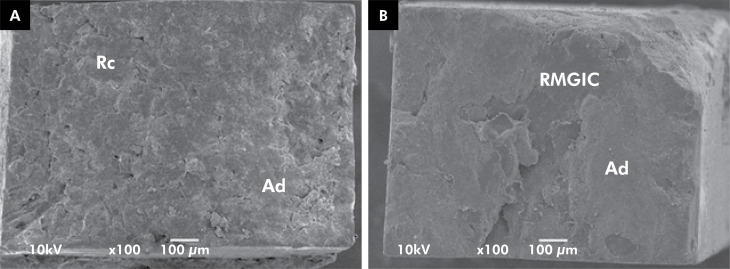
SEM images of fractured specimens representative of the mixed/adhesive failure pattern from: 1A: repair with universal adhesive in the etch-and-rinse mode plus resin composite and 1B: aged resin-modified glass ionomer cement.

**Figure 2:**
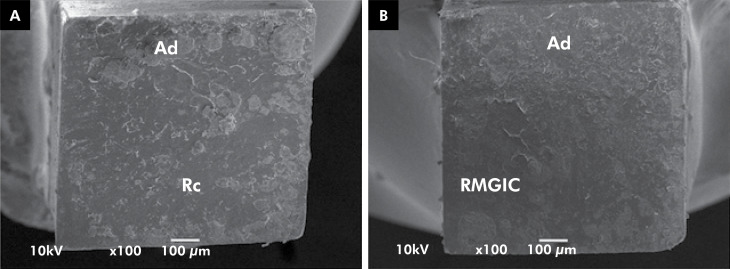
SEM images of fractured specimens representative of the mixed/adhesive failure pattern from: 2A: repair with universal adhesive in the self-etch mode plus resin composite and 2B: aged resin-modified glass ionomer cement.

**Figure 3:**
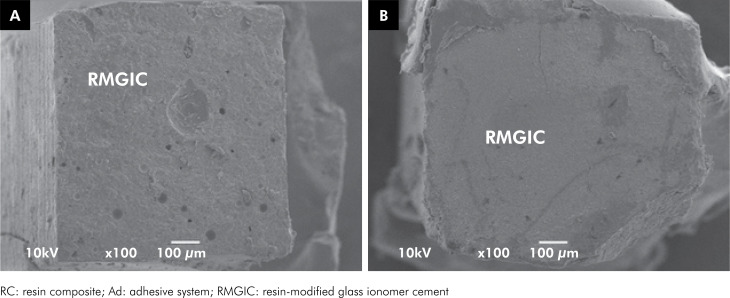
SEM images of fractured specimens representative of the mixed/adhesive failure pattern from: 3A: repair with resin-modified glass ionomer cement and 3B: aged resin-modified glass ionomer cement.

The μTBS means, standard deviations, and distribution of the failure modes for all the experimental groups are shown in Table 2. The highest μTBS values were obtained when the resin-modified glass ionomer cement was repaired using the same material (p < 0.01). In addition, the mode of application of the universal adhesive system did not influence the repair bond strength of the resin-modified glass ionomer cement. Mixed/adhesive failures prevailed in all the groups. This pattern was further confirmed by SEM images (Figures 1, 2 and 3). A higher frequency of cohesive failures was observed when repair was performed with resin-modified glass ionomer cement. Premature failures were more frequent for repair with resin composite when the universal adhesive was used in self-etch mode.

**Table 2:** The microtensile bond strength means (MPa), standard deviations, and distribution of the failure mode for all experimental groups.

Repair protocol	Bond strength	Mixed/Adhesive	Cohesive	Premature
SBU ER+ RC	21.1 ± 7.7^B^	34 (70.8%)	14 (29.2%)	0 (0%)
SBU SE + RC	12.0 ± 7.6^B^	26 (53.1%)	17 (34.7%)	6 (12.2%)
RMGIC	33.7 ± 8.6^A^	34 (50%)	33 (48.5%)	1 (1.5%)

SBU: Scotchbond Universal Adhesive; ER: etch-and-rinse; SE: self-etch; RC: resin composite; RMGIC: resin-modified glass ionomer cement.*Different capital superscript letters indicate statistically significance differences between bond strength values of the repaired groups (p < 0.01).

## Discussion

A recent survey^4^ showed that clinicians prefer repairing defective glass ionomer cement restorations with the same material, followed by resin composites. Therefore, in this study, we investigated the repair potential of resin-modified glass ionomer cement using different adhesive materials. Repair of the resin-modified glass ionomer with the same material resulted in higher μTBS values than those with the resin composite. A previous study^5^ measured the repair bond strength of two resin-modified glass ionomer cements (Fuji II LC and Ketac N100), using either the same resin-modified glass ionomer cement or resin composite as the repair material. The results were material-dependent, indicating that the repair of Ketac N100 with additional Ketac N100 may be clinically unpredictable. However, the repair of Fuji II LC with either Fuji II LC or resin composite would be acceptable. Ketac N100 is a nanofilled resin-modified glass ionomer with a highly packed filler composition (~ 69%), of which approximately two-thirds are nanofillers. The primary curing mechanism is light activation, and no redox or self-curing occurs during setting.^11^ These characteristics could explain why the resin composite bonded better to the aged Ketac N100 than to the same material. Conversely, Fuji II LC and the resin-modified glass ionomer cement used in this study (Riva Light Cure) have very similar compositions. However, it is important to highlight that in contrast to this study, repair procedures in the previous study were not performed in "non-aged" resin-modified glass ionomer cements.

Aging of the glass ionomer cement surface was also shown to be a significant factor influencing the repair bond strength, and increased aging reduced the repair bond strength.^6,12^ Although there is no aging protocol that is considered the gold standard for mimicking the aging of dental materials that occurs in the oral environment, in this study, resin-modified glass ionomer cement was aged by water storage for 14 d followed by thermocycling.^9^ All aged resin-modified glass ionomer cements were roughened using a 320-grit silicon carbide grinding paper, simulating the roughness obtained with a medium diamond bur,^9^ to obtain micromechanical retention before any additional chemical treatments were performed. It has been shown that conditioning for 20 s with phosphoric acid or roughening of the surface followed by acid etching promotes the bond between the aged and new glass ionomer cement.^12^ It was suggested that the exposed glass particles in the aged material could react with the acid in the new material and thus establish a chemical bond.^6^ Scanning electronic microscopy images revealed that after roughening, the resin-modified glass ionomer cement surface appeared relatively rough, with numerous porosities and abraded glass particles. However, no substantial difference was observed when phosphoric or polyacrylic acid was used.^5^ Therefore, in this study, pre-repair treatment using resin-modified glass ionomer cement was performed with phosphoric acid for 20 s. A previous study^7^ found that the repair of an encapsulated glass hybrid restorative system (Equia Forte Fil) with the same material provided lower μTBS values compared to repair with a universal adhesive in the etch-and-rinse mode and resin composite.

It is relevant to note that the bond strength values obtained in the present study were much higher than the previous studies.^5-7^ These, only one study^7^ used the same bond strength test and a protocol for aging the glass ionomer cement surface prior the repair. Despite that these similarities, the repair with Equia Forte Fil was performed after conditioning with polyacrilic acid, but without previous surface roughening, which may explain the contradictory results of this study.

Furthermore, the mode of application of the universal adhesive system did not influence the repair bond strength of the resin-modified glass ionomer cement. Similar μTBS values were obtained when the universal adhesive was used in the etch-and-rinse and self-etch modes. However, a higher number of premature failures was observed when the universal adhesive was used in self-etch mode.

The repair bond strength was measured as the maximum force prior to specimen fracture.^13^ If a large percentage of the specimens are cohesively fractured, few conclusions can be drawn regarding the repair bond strength because the bond strength is usually lower than the cohesive strength. Cohesive failures most commonly occurred when repair was performed with resin-modified glass ionomer cement; however, these failures were not included in the mean bond strength. This finding is in line with previous studies that have tested the repair of resin-modified glass ionomer cements.^5,6^ Bond strength testing of glass ionomer cement to tooth structure frequently results in cohesive failure of the material, and failure stress is probably representative of the strength of the glass ionomer cement itself. The limitations of this study must be addressed. The results are based on immediate repair bond strength values and are limited to the materials used in this study. Further studies are required to evaluate the long-term repair potential of resin-modified glass ionomer cements using the same material or resin composite.

## Conclusions

Repair of resin-modified glass ionomers with the same material appears to be the preferred option to improve bond strength. When resin composite is used for repair, the use of universal adhesive in the self-etch mode simplifies the protocol.
